# Tofacitinib Versus Vedolizumab Among Bio-naive Patients With Ulcerative Colitis: A Real-World Propensity-Weighted Comparison

**DOI:** 10.1093/ecco-jcc/jjae188

**Published:** 2024-12-11

**Authors:** Beatriz Gros, Nathan Constantine-Cooke, Jake Kennedy, Alexander T Elford, Claire O’Hare, Colin Noble, Gareth-Rhys Jones, Ian D Arnott, Charlie W Lees, Nikolas Plevris

**Affiliations:** Edinburgh IBD Unit, Western General Hospital, Edinburgh, UK; Department of Gastroenterology and Hepatology, Reina Sofía University Hospital, IMIBIC, University of Córdoba, Córdoba, Spain; Biomedical Research Center in Hepatic and Digestive Disease, CIBEREHD, Madrid, Spain; Centre for Genomics and Experimental Medicine, Institute of Genetics and Cancer, University of Edinburgh, Western General Hospital, Edinburgh, UK; MRC Human Genetics Unit, Institute of Genetics and Cancer, University of Edinburgh, Western General Hospital, Edinburgh, UK; Edinburgh IBD Unit, Western General Hospital, Edinburgh, UK; Edinburgh IBD Unit, Western General Hospital, Edinburgh, UK; Faculty of Medicine, The University of Melbourne, Melbourne, Australia; Edinburgh IBD Unit, Western General Hospital, Edinburgh, UK; Edinburgh Pharmacy Unit, Western General Hospital, Edinburgh, UK; Edinburgh IBD Unit, Western General Hospital, Edinburgh, UK; Edinburgh IBD Unit, Western General Hospital, Edinburgh, UK; Centre for Inflammation Research, The Queen’s Medical Research Institute, University of Edinburgh, Edinburgh, UK; Edinburgh IBD Unit, Western General Hospital, Edinburgh, UK; Edinburgh IBD Unit, Western General Hospital, Edinburgh, UK; MRC Human Genetics Unit, Institute of Genetics and Cancer, University of Edinburgh, Western General Hospital, Edinburgh, UK; Edinburgh IBD Unit, Western General Hospital, Edinburgh, UK; MRC Human Genetics Unit, Institute of Genetics and Cancer, University of Edinburgh, Western General Hospital, Edinburgh, UK

**Keywords:** Ulcerative colitis, biologics, small molecule, real-world data

## Abstract

**Background and Aims:**

Over the last decade, treatment options for moderate-to-severe ulcerative colitis (UC) have expanded. However, comparative studies between these agents are limited, especially among biologic-naive patients. We aimed to compare the persistence, effectiveness, and safety of tofacitinib and vedolizumab as the first advanced treatment for patients with UC.

**Methods:**

Patients who received either tofacitinib or vedolizumab as their first advanced therapy for UC in NHS Lothian were included. We used inverse probability of treatment weighting. The probability of treatment assignment was calculated via logistic regression using age, sex, UC duration, Montreal extent, C-reactive protein, concomitant corticosteroids, and partial Mayo score at drug commencement.

**Results:**

We included *n* = 158 patients, of whom *n* = 81 (51.3%) received vedolizumab and *n* = 77 (48.7%) tofacitinib. Median follow-up for vedolizumab patients was 3.1 years (interquartile range [IQR] 1.6-4.8) and for tofacitinib patients 1.5 years (IQR 0.3-2.3). The cohort was 59.5% male with a median age of 41.1 years (IQR 31.5-51.8). At 2 years, vedolizumab persistence was superior to tofacitinib (*p* = 0.005). At Weeks 12 and 52, clinical, biochemical, and fecal biomarker steroid-free remission were comparable between groups. Primary nonresponse and secondary loss of response were 9.9% and 17.3% for vedolizumab and 23.4% and 13% for tofacitinib, respectively. The frequency of adverse events was comparable (11 [13.6%] vedolizumab vs 19 [24.7%] tofacitinib, *p* = 0.629).

**Conclusions:**

We found that the persistence and tolerability of vedolizumab were superior to tofacitinib in bio-naive UC, although the rates of clinical and biomarker remission were comparable. These data may help inform the positioning of advanced therapy.

## 1. Introduction

In the last decade, the number of available advanced therapies for ulcerative colitis (UC) has significantly expanded.^1^ However, determining the optimal positioning of these therapies remains a matter of debate. The scarcity of head-to-head randomized control trials (RCTs) has resulted in clinicians relying on network meta-analysis evidence to guide their decisions.^2^ Nevertheless, the inclusion of a substantial proportion of patients with prior biologic experience in these RCTs, coupled with selective and controlled methods employed to ensure high internal validity, restricts the applicability of the findings to a heterogeneous population.^3^ As such, determining first-line therapy remains a major challenge and most guidelines have not been able to prioritize one drug over another.^4^

Regarding indirect comparisons, previous real-world studies have evaluated the differences between ustekinumab and tofacitinib in UC^5,6^ as well as the impact of drug sequencing.^7^ However, these studies primarily focused on patients with prior antiTNF exposure. To the best of our knowledge, real-world data comparing a biologic drug against a small molecule in biologic-naive patients has not been published.

Vedolizumab, an α4β7 anti-integrin drug was approved for the therapy of UC in 2013, while tofacitinib, a Janus kinase inhibitor, received approval in 2017. Both drugs have demonstrated efficacy in inducing and maintaining remission in UC.^8,9^ However, both show a significant decrease in their efficacy when used in patients previously exposed to antiTNF.

At the Edinburgh IBD Unit, both vedolizumab and tofacitinib have been extensively utilized as first-line therapy in UC. The aim of this study was to assess the real-world persistence, effectiveness, and safety of both therapeutic strategies in biologic-naive patients. This provides valuable insights that complement existing clinical trials and real-world evidence, assisting clinicians in making informed decisions regarding the first therapeutic choice in biologic-naive UC.

## 2. Methods

### 2.1. Study design

We performed a retrospective, observational, cohort study performed at 3 sites within NHS Lothian (Royal Infirmary Hospital, Western General Hospital, and St John’s Hospital). NHS Lothian provides universal, free at the point of care healthcare for 916 310 people (2022) in Edinburgh and the surrounding area and has a rigorously validated prevalent population of 10 499 patients with IBD (2019).^10^

### 2.2. Participants

We identified all adult (>18 years old) patients with UC receiving vedolizumab or tofacitinib, as first-line advanced therapy, via pharmacy and electronic medical health records (TrakCare patient management ©InterSystems). Follow-up was until November 1, 2023. Inclusion criteria were a confirmed diagnosis of UC (based on standard criteria) and active disease (clinical symptoms attributed to UC plus C-reactive protein [CRP] >5 mg/L and/or fecal calprotectin [FC] >250 µg/g and/or endoscopically active disease). Patients with Crohn’s disease, IBD-U favoring Crohn’s disease, microscopic colitis, pouchitis, previous biologic or small molecule exposure, patients on immunotherapy or chemotherapy for cancer; patients with solid organ transplant and combination with immunosuppressant drugs were excluded (Supplementary Figure 1). To allow for a more balanced comparison, subjects aged 65 years or older were excluded from both drug groups, given the regulatory guidelines for the use of JAK inhibitors as first-line therapies across immune-mediated inflammatory diseases, which states this class should only be used in this age group when no appropriate alternative exists.^11^

### 2.3. Data collection

Baseline demographic, phenotyping, and follow-up data were collected by reviewing electronic medical records. Data regarding clinical disease activity scores (partial Mayo score); CRP; FC; dose adjustments; steroid prescriptions; hospitalization rates; and adverse events were collected via review of electronic medical records. Available CRP and FC data at baseline, 12 ± 8 weeks, and 52 ± 8 weeks from drug commencement were analyzed. All FC samples were measured using a standard enzyme-linked immunosorbent assay (ELISA, Calpro AS™, Norway).

### 2.4. Definitions

Clinical remission, biochemical remission, and fecal biomarker remission were defined as a partial Mayo <2, CRP ≤5 mg/L, and FC <250 µg/g, respectively. Normal albumin level was defined as ≥36 g/L. Primary nonresponse was defined as ongoing disease activity evidenced by an elevated partial Mayo score (≥2) and elevated biochemical and/or fecal biomarkers (CRP >5 mg/L/FC ≥250 µg/g) from therapy commencement leading to treatment discontinuation within 5 months from drug initiation. Secondary loss of response was defined as initial clinical and/or biomarker remission with subsequent development of an elevated partial Mayo score (≥2) and/or elevated biochemical and fecal biomarkers (CRP >5 mg/L/FC ≥250 µg/g) resulting in treatment discontinuation. Steroid prescription during follow-up was defined as oral prednisone or oral budesonide prescribed at any point after vedolizumab or tofacitinib commencement. Serious adverse events were defined as any event leading to permanent discontinuation of therapy, hospitalization, or death; moderate adverse events were defined as any event requiring temporary discontinuation with all other adverse events defined as mild. An increase of baseline cholesterol needing statin prescription was not considered as an adverse event. Drug intensification was done as per clinician discretion with tofacitinib being increased up to 10 mg *bis in die* (BD) and vedolizumab intensified up to 300 mg every 4 or 6 weeks. The date of drug intensification was recorded. If a patient was known to be lost to follow-up due to the patient transferring to another health board, then these subjects were excluded (*n* < 5).

### 2.5. Primary and secondary outcomes

The primary outcome of this study was drug persistence. Temporary discontinuations of therapy were not considered to calculate drug persistence and only drug withdrawal was considered. Secondary outcomes included primary and secondary loss of response; adverse events; clinical, biochemical, and fecal biomarker steroid-free remission after induction at Weeks 12 and 52.

### 2.6. Statistical analysis

Descriptive statistics are presented as medians with interquartile range [IQR] for continuous variables, and frequencies with percentages for categorical variables. For comparison of nonparametric continuous variables, the Mann–Whitney *U* test was used. For comparison of categorical variables, the chi-squared test was used. Kaplan–Meier survival curves were generated to assess drug persistence. Subjects were censored at drug cessation or last clinical follow-up/death. A per-protocol analysis was used for the effectiveness outcomes. A *p*-value <0.05 was considered significant.

For the adjusted analysis, propensity scores were calculated via logistic regression using age, sex, disease duration, Montreal extent, CRP, concomitant steroid usage, and partial Mayo score at the time of drug commencement. Missing data for CRP (*n* = 1), partial Mayo score (*n* = 7), and Montreal extent (*n* = 1) were imputed using multiple imputation via chained equations with 500 iterations.^12^ The mean for continuous variables and mode for categorical variables were used to calculate singular values for each missing data point. Inverse probability of treatment weighting (IPTW) was used to adjust the treatment groups, and standardized mean difference (SMD) was used to assess covariate balance with <0.1 used as the cutoff for a covariate being balanced. Kaplan–Meier survival curves weighted via IPTW were generated for time to treatment cessation. The Pepe and Fleming test^13^ was used to test for differences between adjusted Kaplan–Meier curves up to 1000 days from treatment commencement. As a sensitivity analysis, subsequent regression adjustment was used. Time-to-cessation between treatment arms was modeled using Cox regression with propensity scores, treatment, and the covariates used to calculate propensity scores. As <5 subjects were found to have extreme propensity scores (<0.1 or >0.9) we have not removed subjects with extreme propensity scores as a sensitivity analysis. Analyses were performed using R (v.4.3.3) with the mice (v.3.16.0), survey (v.4.4-2), table one (v.0.13.2), and adjusted Curves (v.0.11.1) extension packages. The R code used to produce the analyses is available on GitHub (https://github.com/nathansam/VedoVsTofa).

### 2.7. Ethics

This work was considered a clinical audit as all data were collected as part of routine clinical care. Therefore, no written consent or formal ethical approval was necessary as per departmental policy and the Health Research Authority.^14^ Caldicott Guardian approval was granted for data collection and publication (NHS Lothian).

## 3. Results

### 3.1. Study population

A total of *n* = 158 patients met the inclusion criteria, of whom *n* = 81 (51.3%) patients received vedolizumab and *n* = 77 (48.7%) patients received tofacitinib. Median follow-up for patients on vedolizumab was 3.1 (1.6-4.8) years and for tofacitinib 1.5 (0.34-2.3) years. The total cohort consisted of *n* = 94 (59.5%) males and the median age was 41.1 (31.5-51.8) years with a median disease duration of 6.21 (2.56-13.5) years (Table 1).

**Table 1:** Demographic and clinical data for the analyzed cohort at time of drug commencement.

	Vedolizumab (*N* = 81)	Tofacitinib (*N* = 77)	Overall (*N* = 158)	*p*-Value
Age (y)				0.687
Median [IQR]	39.9 [29.5, 52.4]	42.1 [35.0, 50.1]	41.1 [31.5, 51.8]	
Gender				1
Male	48 (59.3%)	46 (59.7%)	94 (59.5%)	
Female	33 (40.7%)	31 (40.3%)	64 (40.5%)	
Disease duration (y)				0.269
Median [IQR]	7.12 [2.99, 14.9]	5.90 [1.56,13.1]	6.21 [2.56, 13.5]	
Extent				0.001
E1	6 (7.4%)	15 (19.5%)	21 (13.3%)	
E2	27 (33.3%)	38 (49.4%)	65 (41.1%)	
E3	47 (58.0%)	24 (31.2%)	71 (44.9%)	
Missing	1 (1.2%)	0 (0%)	1 (0.6%)	
Partial Mayo score				0.020
Median [IQR]	6.0 [4.0, 7.0]	6.0 [5.5, 7.0]	6.0 [5.0, 7.0]	
Missing	5 (6.2%)	2 (2.6%)	7 (4.4%)	
CRP (mg/dL)				0.348
Median [IQR]	3 [1, 7]	3 [1, 9]	3 [1, 7]	
Missing	0 (0%)	1 (1.3%)	1 (0.6%)	
FC (µg/g)				0.475
Median [IQR]	640 [338, 1190]	934 [333, 1134]	775 [332, 1150]	
Missing	18 (22.2%)	9 (11.7%)	27 (17.1%)	
Concomitant corticosteroids				0.790
Yes	49 (60.5%)	44 (57.1%)	93 (58.9%)	
No	32 (39.5%)	33 (42.9%)	65 (41.1%)	

Abbreviations: CRP, C-reactive protein; FC: Fecal calprotectin; IQR, interquartile range.

Disease extension differed between groups, with pancolitis (E3) being predominant in the vedolizumab group (*n* = 47 [58%] vs *n* = 24 [41.2%]) and left-side colitis (E2) in the tofacitinib group (*n* = 27 (33.3%) vs *n* = 38 (49.4%), *p* = 0.001). Partial Mayo score at baseline was statistically different but numerically similar between the vedolizumab group and the tofacitinib group (vedolizumab: 6.0 [IQR 4.0-7.0] vs tofacitinib: 6.0 [IQR 5.5-7], *p* = 0.02). There were no differences in steroid prescription, CRP, or FC at baseline between the 2 groups (Table 1).

### 3.2. Unadjusted outcomes

**Figure 1:**
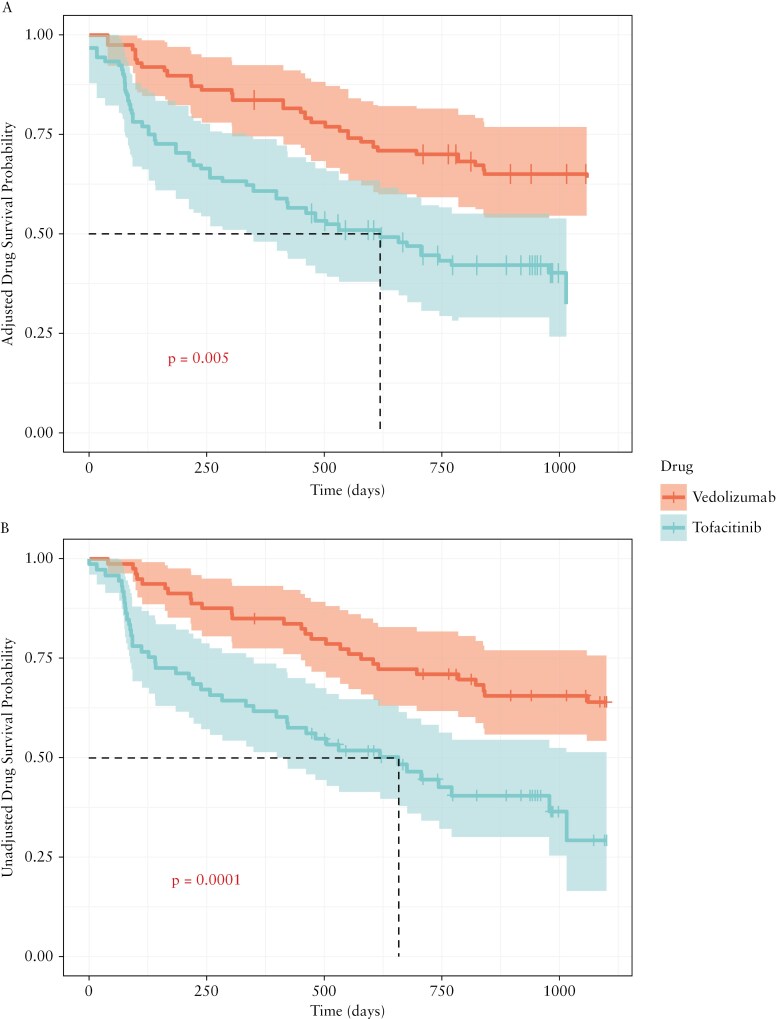
Treatment persistence of vedolizumab and tofacitinib. A, Adjusted via inverse probability of treatment weighting; B, unadjusted.

In the unadjusted analysis, vedolizumab demonstrated significantly better persistence than tofacitinib during follow-up (Figure 1; *p* = 0.0001). Two years after treatment commencement, cumulative survival rates were 71% (95% confidence interval [CI] 62%-82%) and 45% (95% CI 34%-58%) for vedolizumab and tofacitinib, respectively.

**Figure 2:**
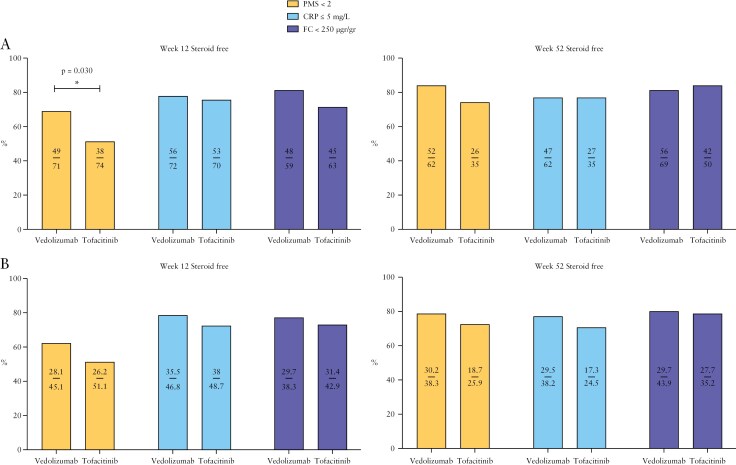
Steroid-free clinical, biochemical, and fecal calprotectin remission at Weeks 12 and 52. A, Unadjusted; B, adjusted. *Statistically significant

At Week 12, steroid-free clinical remission rates were higher in the vedolizumab group when compared with the tofacitinib group (vedolizumab: *n* = 49 [69%] vs tofacitinib: *n* = 38 [51.4%], *p* = 0.030) (odds ratio [OR] 2.11; 95% CI 1.07-4.16). However, no differences were observed in steroid-free biochemical remission rates (vedolizumab: *n* = 56 [77.8%] vs tofacitinib: *n* = 53 [75.7%], *p* = 0.77) or in steroid-free fecal biomarker remission rates (vedolizumab: *n* = 48 [81.4%] vs tofacitinib: *n* = 45 [71.43%], *p* = 0.20) between the 2 groups at Week 12 (Figure 2, A). At Week 52, steroid-free clinical, biochemical, and fecal biomarker remission rates were similar between the vedolizumab and tofacitinib groups (Figure 2, A). Within the full duration of follow-up, the overall withdrawal due to clinical failure was 22 (27.2%) for vedolizumab and 28 (36.4%) for tofacitinib. The median time to drug withdrawal for vedolizumab was 503 days (217-830) versus 163 days (81-466) for tofacitinib. Primary nonresponse and secondary loss of response occurred in *n* = 8 (9.9%) and *n* = 14 (17.3%) patients for vedolizumab; and *n* = 18 (23.4%) and *n* = 10 (13%) patients for tofacitinib, respectively.

There was a significant reduction in FC following 12 weeks of treatment in both the patients on vedolizumab (640 vs 31 µg/g, *p* < 0.001) and tofacitinib (934 vs 93 µg/g, *p* < 0.001). In the tofacitinib group, CRP also significantly dropped by Week 12 (3 vs 1 mg/L, *p* = 0.05), however, there was no significant drop in the vedolizumab group (3 vs 3 mg/L, *p* = 0.584), Supplementary Figure 4.

### 3.3. Steroid prescription, hospitalization, and dose optimization

During follow-up, there were *n* = 15 (18.5%) patients on vedolizumab and *n* = 15 (19.5%) patients on tofacitinib who needed oral corticosteroid prescription (*p* = 0.29). The dose of baseline drug was intensified in *n* = 9 (11.1%) vedolizumab patients (q4w or q6w) and *n* = 22 (28.6%) tofacitinib patients (10 mg BD) (*p* < 0.0001) (OR 2.48, 95% CI 1.67-3.69). Hospitalization was needed in *n* = 6 (7.4%) vedolizumab and *n* = 6 (7.8%) tofacitinib patients (*p* = 0.55). There was 1 colectomy during follow-up in someone who was receiving vedolizumab.

### 3.4. Safety

During follow-up, a total of *n* = 38 (24.1%) adverse events were reported (Table 2). There were no differences in the frequency of adverse events (vedolizumab: *n* = 16 [19.8%] vs tofacitinib: *n* = 24 [31.2%], *p* = 0.11). However, adverse events resulting in temporary discontinuation of the drug occurred in more patients receiving tofacitinib than vedolizumab (vedolizumab: *n* = 1 [1.2%] vs tofacitinib: *n* = 11 [14.3%], *p* = 0.013). A total of *n* = 6 (3.8%) patients withdrew the drug due to adverse events (vedolizumab: *n* = 3 [3.7%] vs tofacitinib: *n* = 3 [3.9%]).

**Table 2:** Adverse events.

	Vedolizumab (*n* = 16)	Tofacitinib (*n* = 22)	Overall (*n* = 38)
All the events			
Mild skin reaction	5	2	7
Chest infection	2		2
Coronavirus (COVID) infection	3	4	7
Allergic reaction	1		1
Joint pain	2	3	5
Nasopharyngitis	1		1
Campylobacter infection	1		1
Coughing	1		1
Otitis		1	1
High Creatin Kinase (CK)		1	1
Shingles		5	5
Headache		3	3
Urinary tract infection		1	1
Increase liver enzymes		1	1
Viral gastroenteritis		1	1
Temporary discontinuation			
Chest infection	1		
Otitis		1	
High CK		1	
COVID infection		5	
Shingles		3	
Viral gastroenteritis		1	
Definitive withdrawal			
Allergic reaction	1		
Joint pain	1	1	
Skin reaction	1		
Shingles		2	

### 3.5. Covariate balance before and after the IPTW

Prior to IPTW, there were significant differences in Montreal disease extent (SMD 0.56), CRP (0.28), and partial Mayo score (0.38) at baseline across treatment groups. For variables used to calculate the probability of treatment assignment, CRP, disease extent, and partial Mayo score were missing for *n* = 1, *n* = 1, and *n* = 7 patients, respectively, and required imputation. After IPTW, the covariate balance between treatment groups was confirmed with <10% absolute standardized differences across all factors considered (Supplementary Figure 2 and 3). Supplementary Table 1 describes the demographics for the adjusted cohort.

### 3.6. Adjusted outcomes

After IPTW, patients receiving vedolizumab continued to demonstrate significantly higher persistence rates when compared with patients receiving tofacitinib (Figure 1; *p* = 0.005). Two years after treatment commencement, cumulative survival rates were 70% (95% CI 59%-81%) and 45% (95% CI 31%-58%) for vedolizumab and tofacitinib, respectively. However, there was no significant difference in adjusted steroid-free clinical, biochemical, and fecal biomarker remission rates between the 2 groups at both Weeks 12 and 52 (Figure 2, B).

## 4. Discussion

In this real-world study, we present data comparing the efficacy and safety between the biologic drug vedolizumab and the small molecule tofacitinib as first-line advanced therapies in UC. Utilizing IPTW to balance the treatment groups, we have been able to produce a comparison adjusted for biases in treatment assignment. The data presented here demonstrates that vedolizumab exhibits better persistence than tofacitinib among biologic-naive patients with over 70% of patients remaining on vedolizumab at 2 years. Although both drugs were able to induce comparable improvements in clinical, biochemical, and fecal biomarker remission at Weeks 12 and 52. During the length of the study, a similar number of courses of steroids were needed in both groups.

The effect of drug positioning on outcomes has been demonstrated in several of the registration trials for both vedolizumab and tofacitinib. Data from the GEMINI 1 trial of vedolizumab showed that 40.8% of biologic-naive patients at Week 6 met the composite outcome of a rectal bleeding score of zero and stool frequency sub-score of one or less. This fell to 24.2% in those previously exposed to a prior biologic.^8,15^ In the same line, data from the OCTAVE Trials 1 and 2 of tofacitinib, demonstrated clinical remission rates at Week 8 of 24.1% for biologic-naive versus 11.4% in those exposed to a prior biologic.^9,16^ Similar results have also been demonstrated in other real-world cohorts. For example, in our previously published vedolizumab experience of 180 patients, we showed that first-line vedolizumab use was the only independent factor associated with clinical remission (hazard ratio [HR] 1.9, 95% CI 1.24-2.91, *p* < 0.01), mucosal healing (HR 2.32, 95% CI 1.11-4.89, *p* = 0.03), and deep remission (HR 3.78, 95% CI 1.55-9.22, *p* < 0.01).^17^ Therefore, it is now accepted that patients with previous exposure to a prior biologic experience have lower rates of efficacy when compared with those receiving the drug first-line. As such performing comparator studies, specifically looking at first-line use is important to help inform drug positioning in clinical practice.

Our data shows a better persistence of vedolizumab over tofacitinib in bio-naive patients, both in the unadjusted and adjusted analysis. Fewer patients in the vedolizumab group also discontinued therapy due to lack of efficacy (27.2% vedolizumab vs 36.4% tofacitinib). This may suggest better effectiveness of vedolizumab over tofacitinib in UC. Others have also shown high persistence rates in vedolizumab responders.^18^ However, in the adjusted analysis, there were no significant differences observed for all effectiveness outcomes (clinical, biochemical, and fecal biomarker remission rates) between the vedolizumab and tofacitinib patients at either Week 12 or 52 (Figure 2). Although, this result needs to be interpreted with caution as not all patients had available data and after IPTW, the overall cohort had an effective sample size of *n* = 104.9 patients (Supplementary Table 1). As such, it may be possible that we were statistically underpowered in detecting differences between the groups. Other reasons for the differences observed in terms of persistence, may be due to variations in onset of action between the drugs. Clinicians may be stopping tofacitinib earlier than they would vedolizumab, which has a perceived longer onset of action Perceived safety differences between the 2 drugs may have also influenced the earlier withdrawal of tofacitinib compared with vedolizumab, thus explaining the higher rates of temporary discontinuation for tofacitinib. Although if this was the case, one would expect the survival curves to begin to converge with time, which is something we did not see. An era effect may have also influenced the vedolizumab group, as clinicians may have had limited further treatment options available to them at the time of prescribing the drug, therefore patients remained on therapy longer. Ultimately, in isolation, persistence is an imperfect assessment of drug effectiveness, as remaining on the drug does not always equate to being in remission.

Both drugs were well tolerated, with only 3 patients in each group needing to discontinue the medication due to adverse events. The reasons for discontinuation were an allergic reaction (*n* = 1), arthralgia (*n* = 1), and skin reaction (*n* = 1) in the vedolizumab group. While shingles (*n* = 2) and arthralgia (*n* = 1) were the reasons for discontinuation in the tofacitinib group. However, we observed a higher likelihood of temporary discontinuation of tofacitinib, primarily due to viral infections. While this risk is well-documented in the use of JAK inhibitors, it is important to acknowledge that daily oral medication is more susceptible to temporary cessation during any infection episode compared with medications administered every 8 weeks intravenously or every 2 weeks subcutaneously because the timing of the infection would need to precisely coincide with the dosing day, which is often impractical to manage. Additionally, none of the patients who had shingles were vaccinated due to the lack of availability of the Shingrex vaccine at the time (now all patients on JAK inhibitor therapy in NHS Lothian receive vaccination routinely), and therefore a number of these viral infections are preventable.

Our study has several strengths including its novelty in focusing solely on biologic-naive patients and employing advanced statistical analysis to adjust the model. Propensity scores were utilized to mitigate potential confounding by indication for vedolizumab or tofacitinib. Following IPTW, baseline covariates between groups were effectively balanced, mirroring the design of a randomized trial. The study also provided detailed clinical data encompassing biochemical and FC results, dose optimizations, adverse events, and reasons for treatment discontinuation throughout the follow-up period spanning 2 years.

Nonetheless, there are some limitations to our study. Primarily, its retrospective nature entails incomplete data for effectiveness outcomes. Furthermore, there might be residual confounding stemming from unmeasured variables that could impact outcomes, such as FC at baseline, which we were unable to control for due to missing data. Also, patients on oral therapies have lower adherence rates compared with intravenous or subcutaneous therapies, even if in our study drug dispensation continued, we cannot capture the real adherence to therapy. Furthermore, due to the real-world retrospective nature of this study, limited endoscopic data was available. It is also important to acknowledge that RCTs represent the best approach for assessing the efficacy and safety of different therapies. However direct comparisons using head-to-head study design are scarce. Our real-world study serves as a complement to RCTs, and provides insights into vedolizumab and tofacitinib effectiveness in biologic-naive patients with UC. Future, head-to-head RCTs are now needed to validate the findings of the real-world comparison studies but also to compare other first-line advanced therapies.

## 5. Conclusion

This real-world study provides valuable insights into the effectiveness and safety of 2 different strategies in biologic-naive patients with UC using vedolizumab or tofacitinib. The findings demonstrate that vedolizumab presents better persistence than tofacitinib. Overall adverse events were comparable although temporary drug discontinuation was more likely to occur with tofacitinib.

## Supplementary Material

jjae188_suppl_Supplementary_Figures_S1-S4_Table_S1

## Data Availability

All data are incorporated into the article and its online supplementary material.
