# Supplementary material for: Tofacitinib Versus Vedolizumab Among Bio-naive Patients With Ulcerative Colitis: A Real-World Propensity-Weighted Comparison
Source: J Crohns Colitis. 2024 Dec 11;19(7):jjae188. doi: 10.1093/ecco-jcc/jjae188 (PMC12260496; doi:10.1093/ecco-jcc/jjae188)
Supplement: jjae188_suppl_Supplementary_Figures_S1-S4_Table_S1 [file jjae188_suppl_supplementary_figures_s1-s4_table_s1.zip › jjae188_suppl_Supplementary_Figures_1-4_Tables_S1/Supplemental material.docx]

**Supplemental material**

**Table S1. Demographic and clinical data for the analyzed cohort after imputation and weighting via propensity scores.**

|  | **Vedolizumab**  **(N=51.42)** | **Tofacitinib**  **(N=53.55)** | **Overall**  **(N=104.97)** |
| --- | --- | --- | --- |
| **Age (years)** |  |  |  |
| Median [IQR] | 36.9 [29.4, 51.9] | 40.3 [31.7, 49.4] | 39.5 [30.7, 50.1] |
| **Gender** |  |  |  |
| Male | 32.4 (63.0%) | 32.9 (61.3%) | 65.2 (62.1%) |
| Female | 19.0 (37.0%) | 20.7 (38.7%) | 39.8 (37.9%) |
| **Disease duration (years)** |  |  |  |
| Median [IQR] | 5.92 [2.65, 10.62] | 5.90 [1.57,13.2] | 5.89 [2.11, 11.8] |
| **Extent** |  |  |  |
| E1 | 6.0 (11.7%) | 6.0 (11.2%) | 12.0 (11.4%) |
| E2 | 24.5 (47.7%) | 26.1 (48.7%) | 50.6 (48.2%) |
| E3 | 20.9 (40.6%) | 21.5 (40.2%) | 42.4 (40.4%) |
| **Partial Mayo score** |  |  |  |
| Median [IQR] | 6.0 [5.0, 7.0] | 6.0 [5.0, 7.0] | 6.0 [5.0, 7.0] |
| **CRP (mg/dL)** |  |  |  |
| Median [IQR] | 3.1 [1.0, 7.0] | 3.0 [1.0, 7.4] | 3.0 [1, 7.4] |
| **FCAL (𝜇g/g)** |  |  |  |
| Median [IQR] | 737 [436, 1042] | 882 [325, 1132] | 777 [411, 1128] |
| **Concomitant**  **corticosteroids** |  |  |  |
| Yes | 32.8 (63.8%) | 34.1 (63.7%) | 66.9 (63.7%) |
| No | 18.6 (36.2%) | 19.5 (36.3%) | 38.1 (36.3%) |

**SUPPLEMENTARY FIGURES**

**FIGURE 1 Flow diagram of inclusion and exclusion criteria**

**FIGURE 2 Covariate balance before and after IPTW. Abbreviations: IPTW, inverse probability of treatment weighting.**

**FIGURE 3 Sensitivity analysis using regression adjustment. Time-to-cessation between treatment arms was modelled using Cox regression with propensity scores, treatment, and the covariates used to calculate propensity scores. Abbreviations: IPTW, inverse probability of treatment weighting.**

**FIGURE 4 Change in (A) faecal calprotectin and (B) CRP between treatment commencement and week 12 (unadjusted). Points denote medians, whilst bars indicate interquartile range. P-values were calculated using paired Wilcoxon signed rank test.**
