# Supplementary figures and images for: Tofacitinib Versus Vedolizumab Among Bio-naive Patients With Ulcerative Colitis: A Real-World Propensity-Weighted Comparison
Source: J Crohns Colitis. 2024 Dec 11;19(7):jjae188. doi: 10.1093/ecco-jcc/jjae188 (PMC12260496; doi:10.1093/ecco-jcc/jjae188)

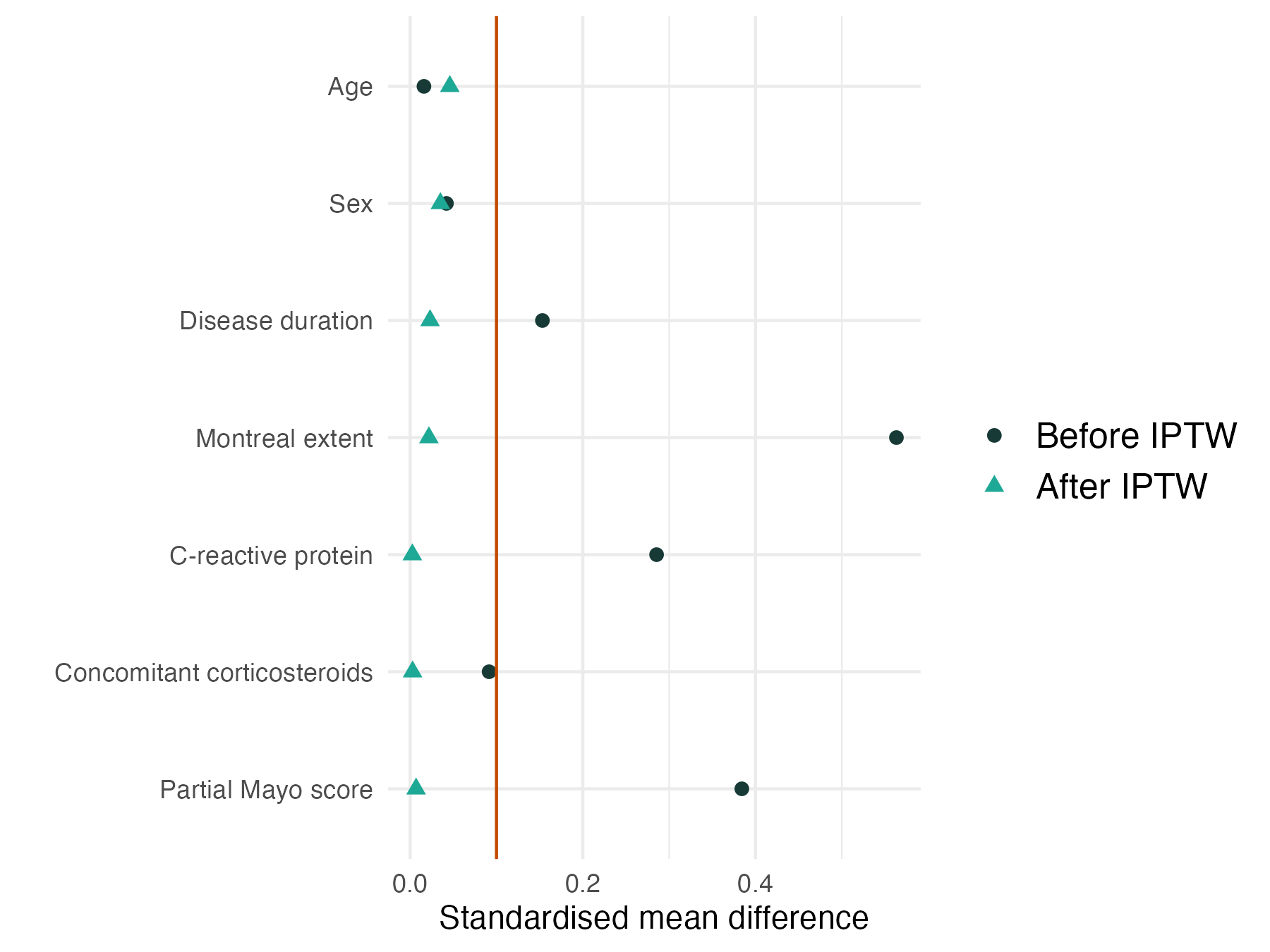

Supplement: jjae188_suppl_Supplementary_Figures_S1-S4_Table_S1 [file jjae188_suppl_supplementary_figures_s1-s4_table_s1.zip › jjae188_suppl_Supplementary_Figures_1-4_Tables_S1/FigureS2.png]

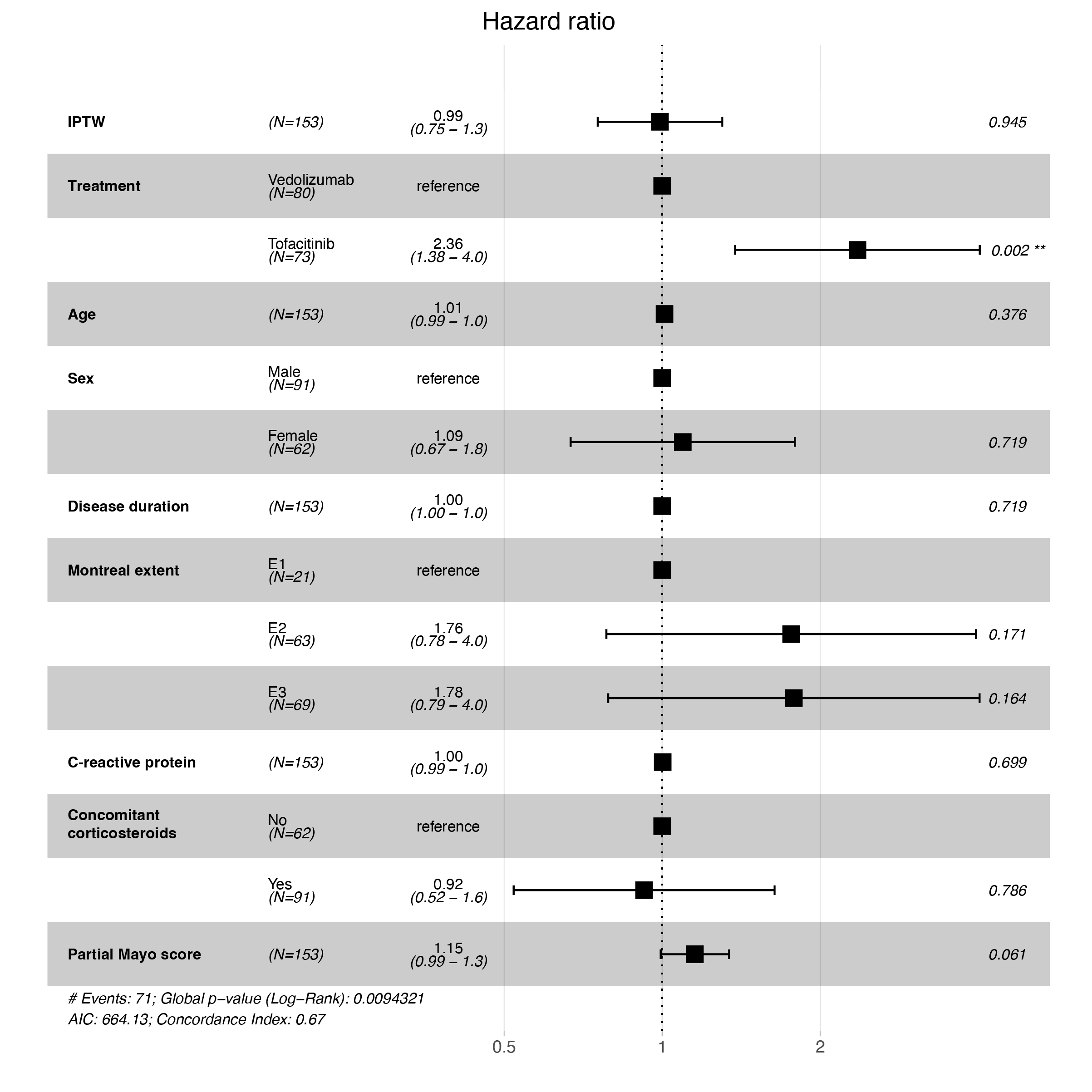

Supplement: jjae188_suppl_Supplementary_Figures_S1-S4_Table_S1 [file jjae188_suppl_supplementary_figures_s1-s4_table_s1.zip › jjae188_suppl_Supplementary_Figures_1-4_Tables_S1/FigureS3.png]

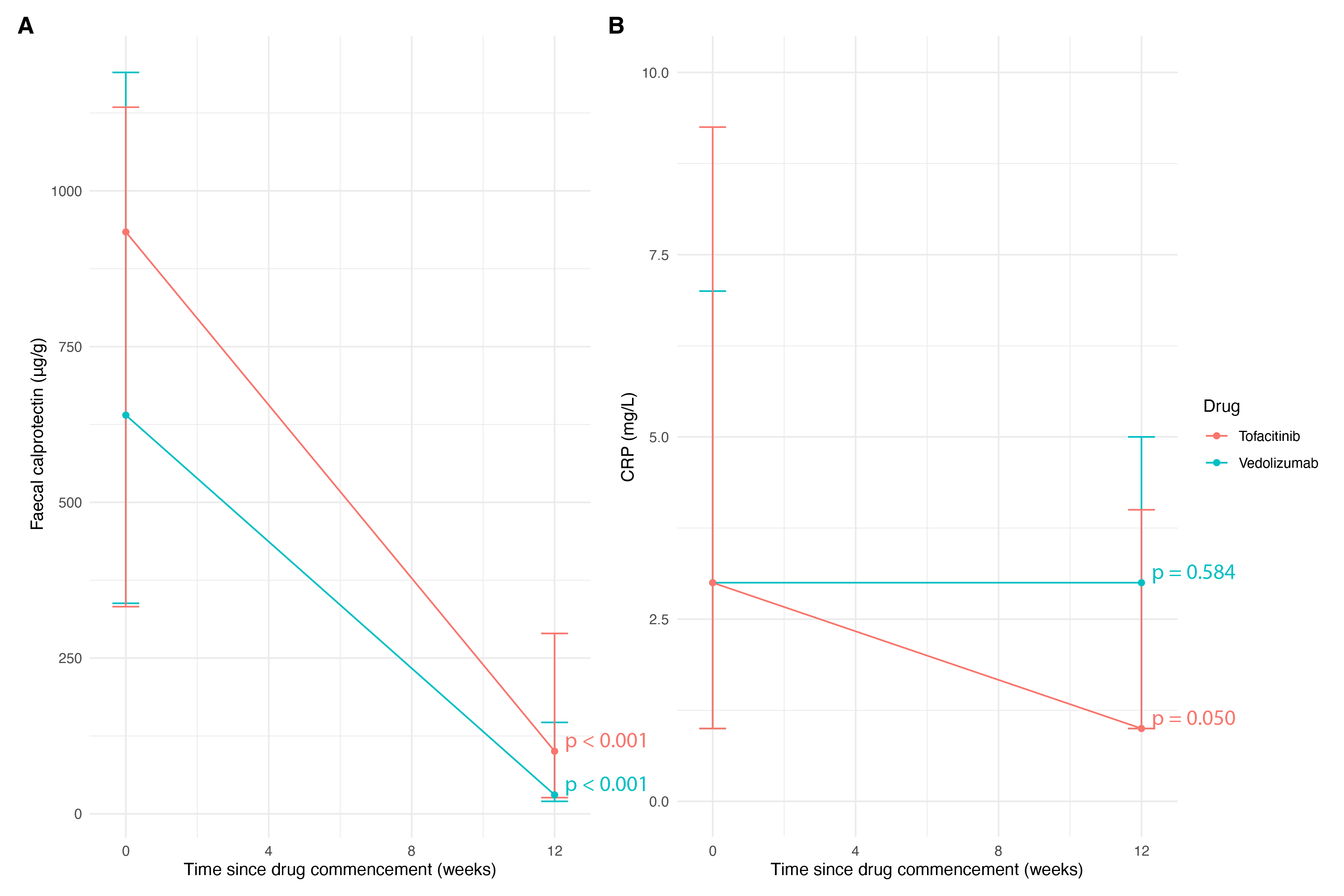

Supplement: jjae188_suppl_Supplementary_Figures_S1-S4_Table_S1 [file jjae188_suppl_supplementary_figures_s1-s4_table_s1.zip › jjae188_suppl_Supplementary_Figures_1-4_Tables_S1/FigureS4.png]

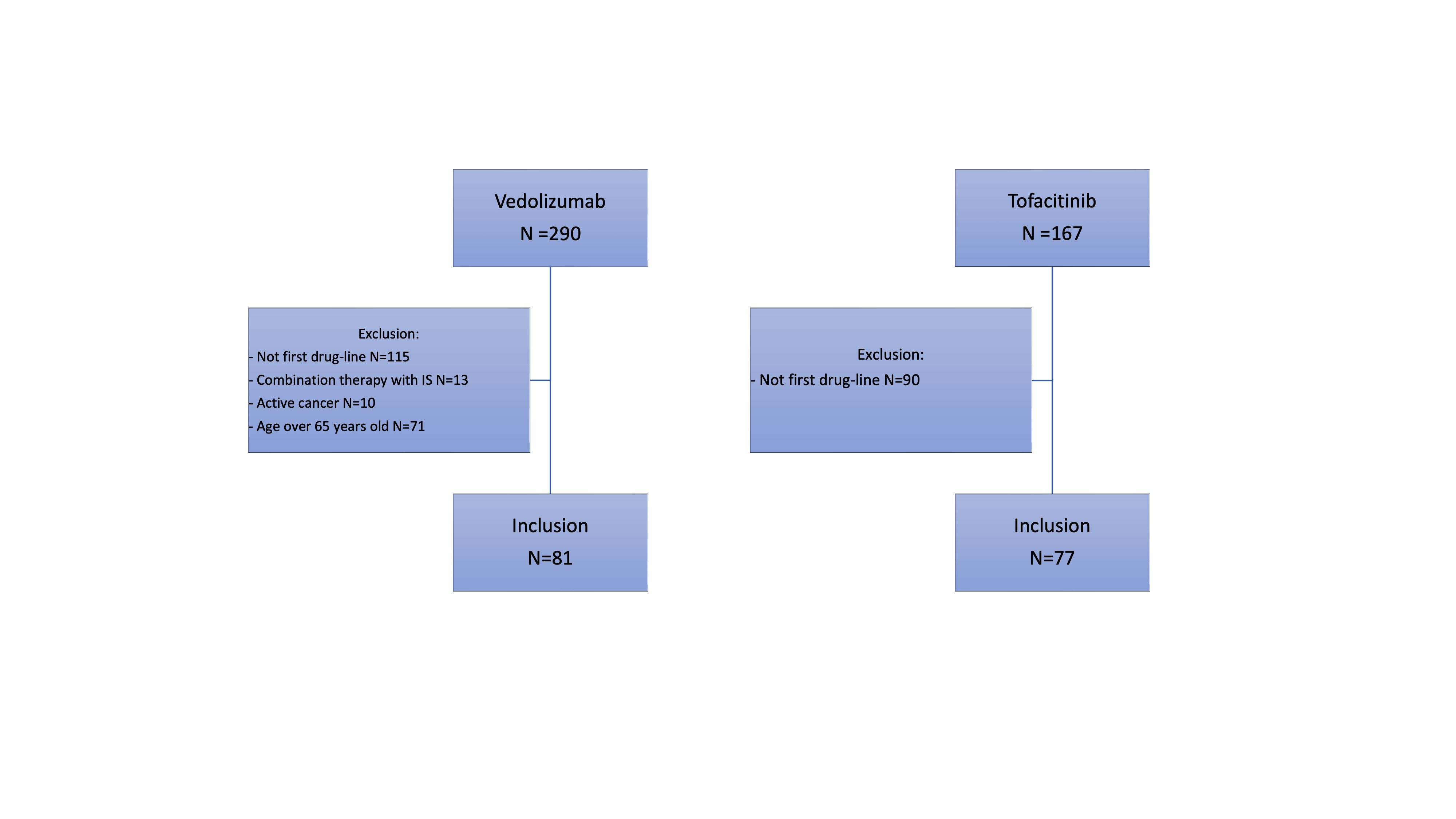

Supplement: jjae188_suppl_Supplementary_Figures_S1-S4_Table_S1 [file jjae188_suppl_supplementary_figures_s1-s4_table_s1.zip › jjae188_suppl_Supplementary_Figures_1-4_Tables_S1/supplementary 1 figure.png]

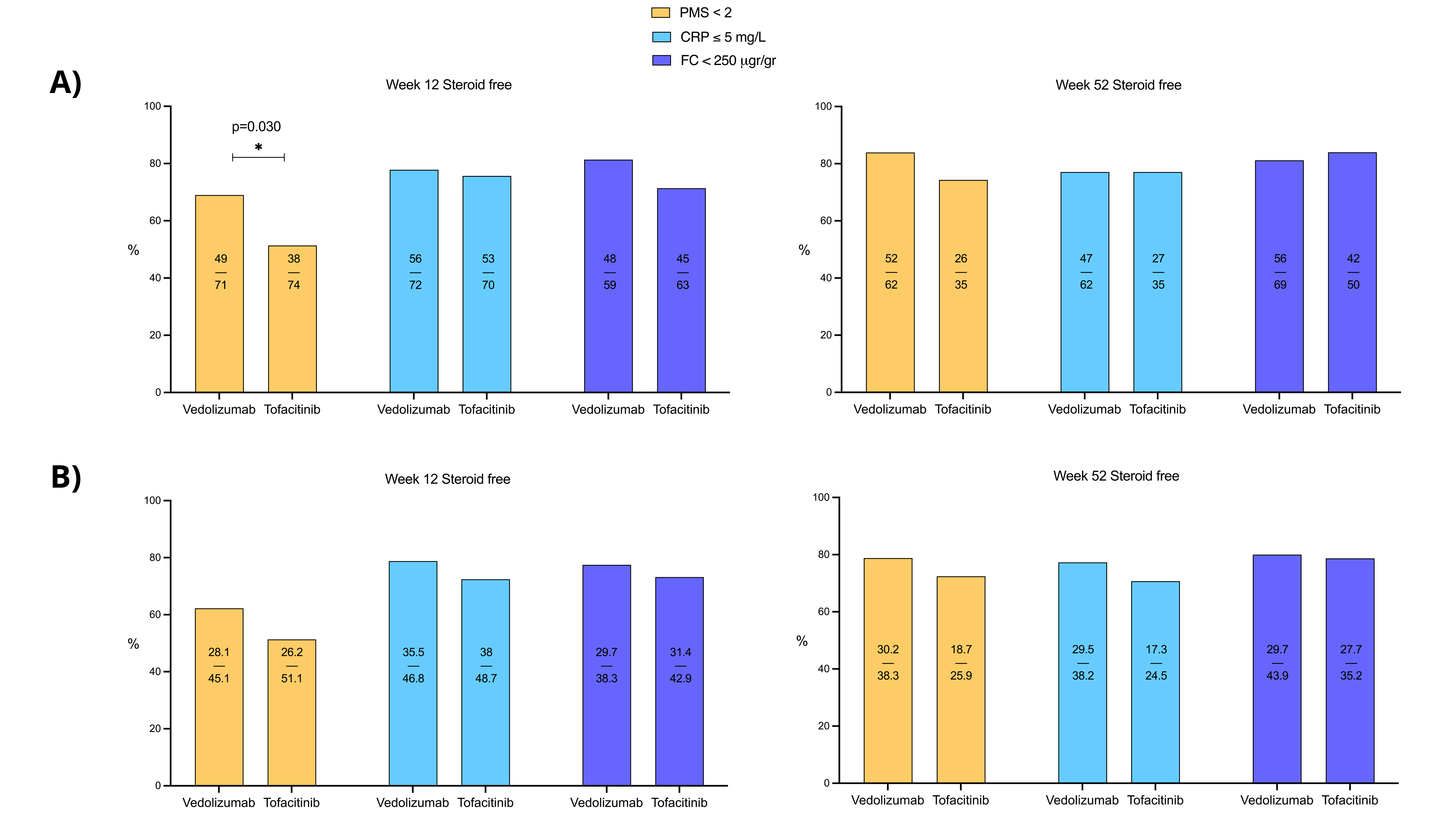

Supplement: jjae188_suppl_Supplementary_Figures_S1-S4_Table_S1 [file jjae188_suppl_supplementary_figures_s1-s4_table_s1.zip › jjae188_suppl_Supplementary_Figures_1-4_Tables_S1/vedo-tofa figure.2png.png]
